# Oral health in the agenda of priorities in public health

**DOI:** 10.1590/S1518-8787.2016050007093

**Published:** 2016-08-26

**Authors:** José Leopoldo Ferreira Antunes, Tatiana Natasha Toporcov, João Luiz Bastos, Paulo Frazão, Paulo Capel Narvai, Marco Aurélio Peres

**Affiliations:** I Departamento de Epidemiologia. Faculdade de Saúde Pública. Universidade de São Paulo. São Paulo, SP, Brasil; II Programa de Pós-Graduação em Saúde Coletiva. Centro de Ciências da Saúde. Universidade Federal de Santa Catarina. Florianópolis, SC, Brasil; III Departamento de Prática de Saúde Pública. Faculdade de Saúde Pública. Universidade de São Paulo. São Paulo, SP, Brasil; IV Australian Research Centre for Population Oral Health. School of Dentistry. The University of Adelaide. Adelaide, Australia

**Keywords:** Dental Caries, Oral Health, Public Health, Review, Historical Article

## Abstract

This study describes the scientific production on oral health diffused in *Revista de Saúde Pública*, in the 50 years of its publication. A narrative review study was carried out using PubMed, as it is the search database that indexes all issues of the journal. From 1967 to 2015, 162 manuscripts specifically focused on oral health themes were published. This theme was present in all volumes of the journal, with increasing participation over the years. Dental caries was the most studied theme, constantly present in the journal since its first issue. Periodontal disease, fluorosis, malocclusions, and other themes emerged even before the decline of dental caries indicators. Oral health policy is the most recurring theme in the last two decades. *Revista de Saúde Pública* has been an important vehicle for dissemination, communication, and reflection on oral health, contributing in a relevant way to the technical-scientific interaction between professionals in this field.

## RESUMO

Este estudo descreve a produção científica sobre saúde bucal veiculada na Revista de Saúde Pública, nos cinquenta anos de sua publicação. Foi realizado estudo de revisão narrativa, utilizando o PubMed como mecanismo de busca que indexa todos os fascículos da revista. De 1967 a 2015, foram publicados 162 manuscritos com foco específico em temas de saúde bucal. Essa temática esteve presente em todos os volumes da revista, com participação crescente ao longo dos anos. Cárie dentária foi o tema mais estudado, marcando presença constante na revista desde seu primeiro fascículo. Doença periodontal, fluorose, oclusopatias e outros temas emergiram antes mesmo do declínio dos indicadores de cárie. Políticas de saúde bucal é o tema mais recorrente nas duas últimas décadas. A Revista de Saúde Pública tem sido importante veículo de divulgação, comunicação e reflexão sobre saúde bucal, contribuindo de modo relevante para a interação técnico-científica entre os profissionais da área.

## INTRODUCTION

Many oral health conditions are recognized as public health problems because of their prevalence, severity, individual and community impact, which entail costs to the health system and the existence of effective methods of prevention and treatment^10^ ^,^ ^59^. Untreated dental caries is considered the most prevalent condition throughout the world^22^; severe periodontal disease is the sixth^21^.

Recognizing the importance of oral and craniofacial diseases on the global burden of morbidity and in association with systemic diseases, treatment costs and the possibility of applying effective measures of promotion and prevention, the 60th World Assembly of the World Health Organization adopted a resolution recommending the member states to increase budgetary appropriations devoted to the control of such diseases and conditions^66^. In Brazil, oral health is one of the three most important reasons for health care demand^20^.

As public health problems, confronting adverse conditions of oral health requires coordinated action on the part of the society, in particular of health services. To guide this action, it is essential to conduct epidemiological studies, of planning and management, and studies on social sciences in health specifically focusing on such conditions in its multiple dimensions.

This understanding permeates the entire history of *Revista de Saúde Pública* (RSP). When celebrating its 40 years, we noted that oral health was one of the main thematic areas of the journal^38^. By studies published in this scientific dissemination vehicle, we can ponder the participation of oral health issues in the evolution of the agenda of priorities in public health.

On the occasion of RSP’s 50th anniversary, the present study aimed at describing the scientific production on oral health issues diffused in the journal throughout its history.

### Published Articles

We carried out a narrative review study. For the retrieval of the published articles, we used the PubMed search database, which indexes all issues of the journal. Two examiners conducted the search independently and resolved conflicts by consensus. The selected articles were classified in subthemes and synthesis for presentation purposes.

From 1967 to 2015, RSP published 162 manuscripts specifically focused on oral health themes, including original articles, theoretical review, reviews, editorials, and previous notes. The Figure shows the distribution of these articles by decades (blocks) and three-year periods (line), showing that RSP approached these themes since its early years and that the concentration of the journal regarding oral health has grown over time.

**Figure:**
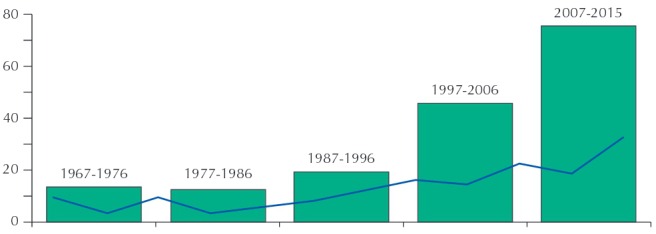
Number of articles on oral health themes published in *Revista de Saúde Pública*, according to decades (blocks) and three-year periods (line). Sao Paulo, SP, Southeastern Brazil, 1967-2015.

### Dental Caries: Prevalence Studies and Inequality

Dental caries was the oral health condition that most motivated studies on the pages of RSP. It was also a constant theme during the five decades of the journal. Sixty-nine articles assessed the prevalence of dental caries in the population in general and in specific groups, inequality in its distribution, preventive resources, and methodological aspects of the forms of measuring the severity.

Already in its early years, dental caries prevalence was described for different cities, having as reference the addition of fluoride in the water supply system^62^ ^,^ ^63^. These studies have contributed to consolidate the conviction that water fluoridation is effective in reducing dental caries indicators, and should, therefore, be extended.

Anticipating the concern with social inequalities in health, pioneering studies addressed differences in the prevalence of dental caries according to social strata. In the first issue of RSP, Souza et al.^56^ compared indicators of the disease among black and white school children; this inequality would be studied again in the journals of 1970^57^ and 1974^7^. Viegas^65^ assessed the incidence of dental caries during pregnancy; Castellanos^7^ studied the prevalence of the disease in orphanages in the city of Sao Paulo. The first study to evaluate socioeconomic differentials in the experience of the disease was published by Yankilevich et al.^67^, having as reference the city of Córdoba, in Argentina, and employing a Marxist-inspired approach, using the concept of social classes.

In the latest period, a decline in the prevalence of dental caries was observed. Articles published in RSP described and analyzed empirical data justifying this observation. Narvai et al.^34^ compared the results of epidemiological surveys of dental caries carried out in the city of Sao Paulo from 1970 to 1996, and concluded that the reduction of dental caries indicators was largely due to the addition of fluoride in public water supply and in dentifrices, as well as the introduction of preventive programs in the public health network. In the same period, Freysleben et al.^17^ compared the presence of dental caries in school children of Florianopolis in 1971 and 1997, concluding that the reduction of the prevalence was real and could not be attributed only to changes in diagnostic criteria.

However, the decline in dental caries prevalence was accompanied by increased inequality of their indicators between the social strata. This phenomenon, referred to as polarization of dental caries experience^35^, was also described and analyzed in the pages of RSP for different age groups^3^ ^,^ ^16^ ^,^ ^55^.

### Original Themes and Methodological Innovations

Methodological aspects of measures of dental caries in the population also motivated pioneering studies on RSP. In 1973, Souza^58^ evaluated a synthetic measure of dental caries experience by simplifying the traditional Decay-missing-filled (DMF) index, which accounts for the decayed teeth, lost due to dental caries and filled (restored). This theme would be explored in the pages of the journal by Guimarães and Guimarães^19^, who proposed original and simplified methodology for measuring prevalence of dental caries and which was applied in subsequent studies^12^.

Analytical techniques widely used in studies on public health also had pioneer application to the area of oral health in the pages of RSP. Linear regression^61^ and logistic regression^40^ were applied to test the association between dental caries experience and behavioral and socioeconomic factors. RSP also innovated by publishing, in 1997, a study with georeferencing of dental caries indicators in the municipalities of the state of Sao Paulo^44^. In 2007, Celeste et al.^9^ published the first article on RSP employing multilevel analysis to relate dental caries indicators in adolescents with the characteristics of individuals and their residential contexts.

Still considering original themes of research in oral health, RSP has published studies that, in time, have had an impact on the prevention of dental caries. Mouthwashes and topical applications of fluoride gel in mouth trays were object of study already in the first volumes of the journal, becoming popular in Brazil^8^ ^,^ ^46^ ^,^ ^64^. Pinto^47^ considered the hypothesis of the addition of fluoride to common salt and underlined the conditions under which the measure could be considered a complement to water fluoridation in the Brazilian context. Peres et al.^42^ described the addition of sugar in syrups and oral drug solutions, with potential damage to children’s oral health. The participation of pediatricians^51^ and community health agents^15^ in promoting children’s oral health also promoted original research on RSP.

The aging of the population, a theme so important to contemporary public health, motivated studies on oral health in RSP in different periods. Dental caries, periodontal disease, dental loss, and prosthetic use were discussed, in 1992, in a study identifying the need for specific odontological policies and programs for older people^50^. Still focused on this age group, several studies have addressed the data of epidemiological surveys carried out in Brazil in recent decades. Singh et al.^54^ analyzed obesity and dental loss in older people and showed that these conditions relate differently between the sexes. Martins et al.^30^ studied the relationship between oral health conditions and housing, individual characteristics, and behavior in a study that included more than 5,000 older people of Brazil.

Figueiredo et al.^14^ evaluated the masticatory capacity of adults in Florianopolis, and Ribeiro et al.^49^ described the dental loss of adults and focused the preservation of functional teeth and reduced dental arch as alternatives to prosthetic treatment. Still regarding the latest period, articles on other original themes were published, which are still reverberating in professional field and have given rise to new approaches such as self-perception of oral health of teenagers^41^ and adults^27^, and the manifestation of lesions of the oral mucosa in patients with HIV/AIDS^45^.

The welfare of professionals working with oral health have also motivated studies. Regarding the decrease in risk of infection of these professionals, Martins and Barreto^32^ described prevalence of vaccination for hepatitis B virus among dentists according to area of expertise, while Garcia and Blank^18^ found the adequacy of occupational post-exposure conducts to biological material for oral health workers. Nunes and Freire^36^ evaluated the quality of life of public health dentists and found low values in physical and psychological domains and high in social relations and environment domains.

### Emerging Themes

Even before the decline of dental caries indicators, other oral health themes emerged as public health problems, requiring action of health authorities and health services. In particular, several studies of RSP focused on distribution, forms of measurement, associated factors, and consequences of malocclusion, gingival inflammation, and periodontal diseases, conditions widely prevalent.

Tomita et al.^60^ pointed the relationship between sucking habits and severity of occlusion such as open bite, dental crowding, and cross bite. The theme would return to the pages of the journal in 2007^39^ and 2013^53^. In 1969, the need for gingival treatment was the subject of RSP^6^. Periodontal disease, gingival bleeding, and dental calculus were themes of the journal, including evaluating the possible association between their manifestation in pregnant women and the birth of children with low weight^11^.

Dental fluorosis, another emerging theme on the agenda of priorities in public health, was present in the pages of RSP in the last two decades of its 50 years. Studies with different methodological schemes assessed its prevalence and its perception by the population as well as the related beliefs and attitudes. Lima and Cury^24^ measured fluoride intake of children by water and dentifrice. Subsequent studies have evaluated the concentration of fluoride in several foods and bottled water for sale.

Oral cancer and orofacial clefts are less prevalent conditions; but their severity justifies the inclusion on the agenda of priorities in public health. These themes are also present in RSP, in the latest period. Loffredo et al. estimated the incidence of oral clefts in Brazil^25^ and carried out a case-control study^26^ pointing heredity and pollution as the main risk factors. Oral cancer has been studied as to its risk factors^28^ ^,^ ^29^ and inequalities of gender and race^2^.

The planning of dental services for patients requiring special care has also been the object of studies published in RSP. Oliveira et al.^37^ studied the dental care aimed at children and adolescents with Down syndrome, and underlined the importance of the orientation of health professionals who take care of these patients in order to provide full care. Elizondo et al.^13^ analyzed the dental care aimed at patients with HIV/AIDS in Mexico by examining their perception regarding the persistence of stigma on the part of professionals.

### Oral Health Policies

The study on oral health policies stood out in the last two decades. About a third of the articles published in RSP in this period addressed oral health policies and related issues of the planning and management of dental services. Such studies proposed and evaluated programs such as fluoridation of the water supply network, preventive procedures and of oral health promotion, the importance of the auxiliary professionals composing oral health teams in direct provision of services, and the dental care in public and private networks.

Even before the Brazilian Unified Health System (SUS) was deployed, Vitor Gomes Pinto^48^ published an article on RSP scaling the treatment needs and human resources in the dental area in order to foster the implementation of a program of basic oral health services of national range. In the following years, the dental care in SUS has been evaluated regarding its extent and effectiveness.

Lacerda et al.^23^ and Martins et al.^31^ evaluated factors associated with self-perception of need to visit the dentist such as pain of dental origin and difficult to chew. Baldani et al.^4^ analyzed the provision of public dental services in Paraná and identified an expansion in oral health actions in previous years, with a pro-equity trend in the provision and use of dental services in primary health care. Antunes and Narvai^1^ also evaluated favorably the expansion of dental care in the public network after the implementation, on the part of SUS, of Family Health Strategy and of Dental Specialties Centers. Peres et al.^43^ documented the reduction in inequalities between socioeconomic strata when using dental services, comparing data collected in 2003 and 2008 for National Research by Residential Samples. Moysés et al.^33^ analyzed the surveillance policy of oral health in force in Brazil, highlighting, on one hand, expressive progress and, on the other, obstacles and difficulties still present.

Camargo et al.^5^ evaluated the use of dental services in pre-scholar children, distinguishing the reason that led to the consultation. By this strategy, they could identify factors associated with consultations aimed at routine evaluation and to solve problems. They concluded that the rate of use of dental services of pre-scholar children is still lesser than that of medical consultations (childcare) and also that, besides the socioeconomic condition, maternal behaviors have an important role for using routine dental services.

On the occasion of the ten-year anniversary of the *Programa Brasil Sorridente* (Smiling Brazil Program), Scherer and Scherer^52^ have focused the changes at work in oral health in primary health care, identifying progress achieved and challenges still persistent at work in oral health at this level of care. According to the authors, the public network professionals tend to reproduce the dominant biomedical model, requiring continued efforts of management, training, and permanent education for understanding the dynamics of the work in order to get significant changes to local realities.

### Final Considerations

RSP has been an important vehicle of communication of scientific knowledge in the field of collective oral health. Since its first issue, and in all its volumes, the journal has been publishing search results about oral health themes of interest to public health, contributing to scientific reflection, professional training, and health planning. The Brazilian Ministry of Health recognized the importance of the journal for the scientific communication in the field of oral health, choosing it to publish, in 2013, a supplement to studies describing and analyzing the results of the National Survey on Oral Health, the epidemiological survey, also known as SBBrasil 2010.

Dental caries has held a prominent position in the published articles. It was also the study on dental caries that, in part, promoted the discussion of methodological innovations, both in terms of its evaluation in epidemiological investigations, and in statistical techniques of analysis. Over time, other harms to oral health, such as adverse periodontal conditions,fluorosis, malocclusions, and oral cancer, were explored according to varied theoretical-methodological perspectives and linked to the Brazilian context.

The growth of publications in the thematic area of policies, planning, management, and evaluation of oral health services sought to answer, among other priorities, the problem represented by dental caries, including demands for qualified services to handle its direct demonstration or more severe consequences. Publications diffused in RSP expose a set of major transformations in scientific thought and valuation of the oral health theme.

The synthetic description of these studies provides identifying the involvement of different oral health themes in the evolution of the agenda of priorities in public health. The journal has been employed by researchers in the field as an instrument of dissemination, communication, and reflection of scientific knowledge, contributing in a relevant way to education and technical, scientific, and professional interaction.
